# Human umbilical cord mesenchymal stem cells accelerate and increase implant osseointegration in diabetic rats

**DOI:** 10.1590/1678-7757-2022-0375

**Published:** 2023-03-27

**Authors:** Mefina KUNTJORO, Nike HENDRIJANTINI, Eric Priyo PRASETYO, Djoko LEGOWO, Ratri Maya SITALAKSMI, Bambang AGUSTONO, ARI Muhammad Dimas Aditya, Guang HONG

**Affiliations:** 1 Universitas Airlangga Faculty of Dental Medicine Department of Prosthodontic Surabaya Indonesia Universitas Airlangga, Faculty of Dental Medicine, Department of Prosthodontic, Surabaya, Indonesia.; 2 Universitas Airlangga Faculty of Dental Medicine Department of Conservative Dentistry Surabaya Indonesia Universitas Airlangga, Faculty of Dental Medicine, Department of Conservative Dentistry, Surabaya, Indonesia.; 3 Universitas Airlangga Faculty of Veterinary Medicine Surabaya Indonesia Universitas Airlangga, Faculty of Veterinary Medicine, Surabaya, Indonesia.; 4 Tohoku University Graduate Scholl of Dentistry Liaison Center for Innovative Dentistry Aoba-Ku Sendai Japan Tohoku University, Graduate Scholl of Dentistry, Liaison Center for Innovative Dentistry, Aoba-Ku, Sendai, Japan.

**Keywords:** Umbilical cord, Mesenchymal stem cells, Dental implants, Osseointegration, Diabetes mellitus

## Abstract

**Objective:**

This study was conducted to assess the effect of hUCMSCs injection on the osseointegration of dental implant in diabetic rats via Runt-related Transcription Factor 2 (Runx2), Osterix (Osx), osteoblasts, and Bone Implant Contact (BIC).

**Methodology:**

The research design was a true experimental design using Rattus norvegicus Wistar strain. Rattus norvegicus were injected with streptozotocin to induce experimental diabetes mellitus. The right femur was drilled and loaded with titanium implant. Approximately 1 mm from proximal and distal implant site were injected with hUCMSCs. The control group was given only gelatin solvent injection. After 2 and 4 weeks of observation, the rats were sacrificed for further examination around implant site using immunohistochemistry staining (RUNX2 and Osterix expression), hematoxylin eosin staining, and bone implant contact area. Data analysis was done using ANOVA test.

**Results:**

Data indicated a significant difference in Runx2 expression (p<0.001), osteoblasts (p<0.009), BIC value (p<0.000), and Osterix expression (p<0.002). In vivo injection of hUCMSCs successfully increased Runx2, osteoblasts, and BIC value significantly, while decreased Osterix expression, indicating an acceleration of the bone maturation process.

**Conclusion:**

The results proved hUCMSCs to accelerate and enhance implant osseointegration in diabetic rat models.

## Introduction

Diabetes is a chronic systemic disease and one of the biggest public health problems in the world. People with uncontrolled diabetes are more prone to tooth loss because of periodontal problem, decreasing the quality of life due to disturbances in the masticatory system. Edentulous people will have unsuccessful dietary management, which is essential to maintain blood sugar levels in the body.^1 , 2^ One way to overcome this issue is by making dentures. There are two types of dentures, removable and fixed. Currently, the first choice for fixed denture is dental implant,^3^ since it has many advantages, such as preservation of dental tissue, bone, improves mastication almost like natural teeth and resistant to caries.^4^ On the contrary, people with uncontrolled diabetes have poor metabolic conditions that can affect the osseointegration process of dental implants. A previous study showed that experimental diabetic rats had a decrease in Bone Implant Contact (BIC) up to 50% and did not reach normal value until the 80^th^ day post implant placement. Moreover, the volume of bone around the implant body also decreased by 50%. These conditions may impact the failure and prolonged dental implant osseointegration process.^5^

Osseointegration is a term used to describe the integration between bone and implants.^6^The osseointegration mechanism occurs when direct contact between the bone and implant body take place. To determine the success of osseointegration process, histological evaluation and histomorphometry can be carried out on experimental animals by examining BIC. The success rate of dental implants in patients without systemic diseases is between 90 and 95%, 10 years post-insertion.^7^ However, in patients with systemic diseases such as uncontrolled diabetes, the success rate decreases especially in the first 2 to 4 weeks after implant placement.^8^

Several studies have been carried out to overcome chronic diabetes complications using stem cells to improve cell regeneration. Recently, Human Umbilical Cord Mesenchymal Stem Cells (hUCMSCs) are widely used because they are multipotent, non-hematopoietic, have paracrine mechanism, can self-repair and differentiate into other cells, such as osteoblasts, adipose tissue, and chondroblasts.^9^hUCMSCs are abundant source, painless collection with no ethical restraint, and have minimal immunogenicity. Compared with other source of stem cells such as bone marrow (BM) or peripheral blood, hUCMSCs have more advantages. hUCMSC can increase the formation of new blood capillaries so that blood circulation in ischemic areas increases.^10^Furthermore, hUCMSCs were applied to treat severe pulmonary arterial hypertension and increased activity of regeneration and anti-inflammation properties, improving clinical parameter of three year old females.^11^ hUCMSCs also significantly improves IL-4, IL-6, and IL-10 expressions, reduce the cytokine storm, and modulates NK cells from severely ill COVID-19 patients.^12^ The injection of hUCMSCs was examined in the osteoporotic mandible and showed significant results in increasing bone density.^13^ The use of hUCMSCs in accelerating the osseointegration process of implants in diabetic patients was never done.

Several markers can determine the osseointegration process, such as Runt-Related Transcription Factor 2 (Runx2), Osterix (Osx), osteoblasts, and BIC. The aim of this study was to determine the effect of hUCMSCs on Runx2, Osx, osteoblasts, and BIC as essential markers to examine the acceleration of osseointegration process of dental implants after hUCMSCs injection in diabetic rat model.

## Methodology

### Ethics approval

All the experiment involving animals were performed in accordance with relevant guidelines and regulations. This animal study was approved by the Animal Research Committee of the School of Veterinary Medicine, Universitas Airlangga (Permit number: 2.KE.152.09.2018) considering the minimal animal model. The study was reported according to ARRIVE guidelines. All experiments involving humans were performed in accordance with relevant guidelines and regulations. Human umbilical cord was obtained from Caesarean delivery. The donor signed the written informed consent and the procedure was approved by the Medical Ethics Committee of Dr Soetomo, General Hospital, Surabaya, Indonesia (Permit number: 547/Panke. KKE/IX/2017)

### Study design

The study design used was true experimental on animals with Randomized Post-Test Only Control Group Design.

### hUCMSCs preparation

Umbilical cord tissue was cleaned and cut to obtain the Wharton’s jelly, which was then dissected into small pieces and cultured using enzyme digestion method until cells were obtained. Cells were resuspended and transferred into a culture dish, then observed under inverted microscope. To confirm MSCs, viable cells were analyzed by flow cytometry. Isolation and culture of the hUCMSCs until passage 6, then 500,000 cells were injected in the implant site. The flowcytometry was performed in passage 4 and confirmed Mesenchymal Stem Cells with positive CD 90, CD 105, CD 73, and negative CD 34, CD 45.^14 - 16^ Cells were cultured in 〈 *minimum essential medium* (MEM) (Gibco BRL, Gaithersburg, MD, USA), *Fetal Bovine serum* (FBS) (Gibco BRL), planted in a 100 mm tissue culture plate (Iwaki, Asahi, Japan) under normoxia conditions (CO_2 _5%), and incubated at 37°C.^17^ Gelatin solvent, which is non-toxic and biocompatible, was used as hUCMSCs scaffold.^18^Conical tubes containing 30 µL hUCMSCs in gelatin were prepared for the injection.

### Sample criteria

The amount of sample size was obtained from the Lemeshow formula, using the data from pilot study. This study was carried out using male *Rattus norvegicus* strain, from 8 to 10 weeks and 150 to 200 grams, healthy according to the experimental animal production criteria, and with high levels of fasting blood sugar–more than 300 mg/dl tested using a portable device glucose test (AccuCheck Performa, Roche, Indonesia) from tail vein (vena lateralis). Diabetic model was induced with Streptozotocin (STZ) 20 mg/kg BW for 5 consecutive days. Freshly prepared STZ (Bioworld, Ohio, USA) dissolved in buffer citrate 0.05 M, pH 4.5 was administered intraperitoneally at 20 mg/kg.^19^ Blood glucose measurement of each animal was measured and recorded ( Table 1 and Table 2 ).

**Table 1:** Blood Glucose Level Measurement (mg/dL) before and after STZ

Sample No.	Before STZ	After STZ	Randomization
1	92	320	T2 (5)
2	89	415	C1 (2)
3	93	375	T1 (4)
4	95	475	T2 (1)
5 **	99	-	
6	84	460	C2 (3)
7	79	482	C2 (7)
8	83	440	C2 (2)
9	96	456	T1 (5)
10 **	88	-	
11	95	411	C2 (5)
12	91	389	T1 (7)
13	90	346	C1 (4)
14	89	469	T1 (3)
15	94	374	C1 (6)
16	92	432	C1 (1)
17	77	458	T2 (4)
18	93	369	C2 (6)
19	86	428	T2 (7)
20	90	445	T2 (6)
21	95	476	C1 (3)
22 **	99	-	
23	84	438	C1 (7)
24	89	497	C2 (1)
25	97	348	T1 (2)
26	78	477	T1 (6)
27	85	453	T1 (1)
28 **	93	-	
29	87	464	T2 (2)
30	95	486	C1 (5)
31	92	490	T2 (3)
32	95	377	C2 (4)

Note : ** indicated animal died after STZ injection intraperitoneally

**Table 2:** Blood Glucose Level Measurement (mg/dL) before implant placement and every week until termination day

Sample No. in each group	Implant placement	1^**st**^ week	2^**nd**^ week	3^**rd**^ week	4^**th**^ week
**C1**					
1	427	467	Hi **	-	-
2	390	421	433	-	-
3	388	434	424	-	-
4	354	398	450	-	-
5	465	427	472	-	-
6	444	497	Hi **	-	-
7	398	489	473	-	-

**C2**					
1	474	434	421	478	455
2	425	480	472	435	Hi **
3	394	435	488	421	412
4	325	396	408	Hi **	462
5	422	433	477	464	Hi **
6	478	455	463	438	474
7	386	423	396	365	415

**T1**					
1	469	483	421	-	-
2	387	379	394	-	-
3	452	485	436	-	-
4	375	394	Hi **	-	-
5	463	494	498	-	-
6	468	423	478	-	-
7	422	446	453	-	-

**T2**					
1	351	372	423	410	452
2	434	479	455	487	Hi **
3	491	493	464	488	475
4	458	432	423	466	474
5	412	384	396	436	453
6	466	426	480	423	444
7	476	425	399	448	466

Note : ** indicated blood glucose level > 500 mg/dL

### Implant specification

The implant (Titanium grade 1) is cylindrical with 1 mm diameter and 2 mm height, machining by CAD/CAM (Yoshimi Inc. Osaka, Japan).

### Number of sample and sample group

In total, 28 Wistar rats were divided into 4 groups (7 rats each). C1 was the implant group and terminated after 2 weeks, C2 was the implant group and terminated after 4 weeks, T1 was the implant group with hUCMSCs injection and terminated after 2 weeks, while T2 was the implant group with hUCMSCs injection and terminated after 4 weeks.

### Implant placement

**Figure 1:**
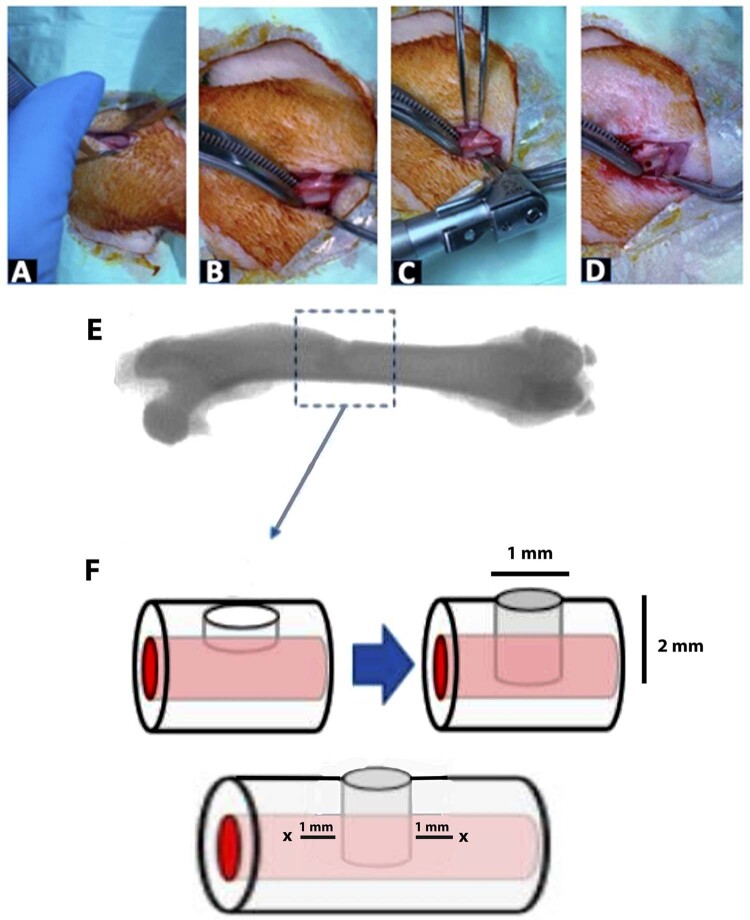
Graphical procedure of how the implant placement was performed. A. Incision was done on the dorsal side of femur. B, C: Osteotomy was done 7 mm from the distal femur edge according to the implant dimension (implant axis), alongside with saline irrigation. D: The implant was placed into the osteotomy site and pushed until it aligned with the femoral bone surface. E: Osteotomy site on the right femur bone of Wistar rats. F: Scheme on sequential the implant placement. X shows the hUCMSCs injection site, 1 mm from the proximal and distal

Before anaesthesia procedures, rats were fasted for 8 hours before implant placement. Ketamine 10% 1 cc and Xylazine 1 cc were injected intramuscularly. The osteotomy area was only located on the right femur, which was shaved and disinfected with Povidone Iodine 10% (Betadine, Indonesia). All the surgical instruments and implants were sterilized with autoclave. A 10 mm incision was performed layer by layer (skin, subcutaneous tissue, muscle, and periosteum) on the dorsal femur’s surface towards the bone surface. Drilling using a bur of diameter 1 mm, K1 drill (Denstply Sirona, Tokyo, Japan) was performed at speed 800 rpm and torque 20 N (NSK Dental Implant Motor non optic Surgery System, Japan), 7 mm from the distal femur edge according to the implant dimension (implant axis), alongside saline irrigation. Before implant placement, the implant bed was irrigated with saline, then the implant was placed into the hole and pushed until it aligned with the femoral bone surface.^20^ For hUCMSCs administration, the bone was perforated intraosseous at 800 rpm speed and 20 N torque, 1 mm from the implant at its proximal and distal side using Stabident (Henry Schein, USA). The hUCMSCs were injected 30 µL in each perforated bone using syringe 27 G for the treatment group (T1 and T2), while control group (C1 and C2) received only gelatin. Suturing on the muscle and skin was performed in layers using 4-0 Polyglycolic Acid Braided Synthetic Absorbable Suture (Surgifit, Busan, Korea). After implant placement, Sulfadiazine Trimethoprim antibiotics (Colibact, Sanbe, Indonesia) 20 mg/ kg body weight intramuscular and Phenylbutazone analgesics (Phenylject, TMC, Indonesia) 20 mg/ kg body weight intramuscular were administered. Figure 1 shows the diagram of the implant placement.

After receiving treatment, all animals presented limited movement for 3 days. However, no lack of appetite nor weight loss or death were observed. After 2 and 4 weeks, the rats were euthanized using perfusion technique (1-cc Ketamine 10% and 1-cc Xylazine, intramuscular). The area of interest was cut at 0.5 mm radius from outer implant margin. The specimen was soaked into 10% buffered formalin for a week, continued with 10% ethylene diamine tetra acetic acid (EDTA) as much as 50 times volume of the specimen’s volume.

### Immunohistochemistry

After deparaffination and rehydrate tissue section, the specimens were washed twice in buffer (Biogear). To reduce non-spesific background staining due to endogenous peroxidase, slides were incubated in Hydrogen Peroxide Block (Thermo Scientific, USA) for 10 minutes, then washed four times in buffer (Biogear). Ultra V Block (Thermo Scientific, USA) was applied and then incubated for five minutes at room temperature to block non-spesific background staining. Primary antibody *(Runx2 monoclonal antibody SC101145 Santa Cruz Biotechnology, USA; Osterix* [ *EPR21034* ] *ab 209484, Abcam, USA)* was applied and incubated at room temperature from around 25 to 30 minutes, then washed four times in buffer (Biogear). Primary Antibody Enhancer (Thermo Scientific, USA) was applied and incubated for 10 minutes at room temperature, then washed four times in buffer (Biogear). HRP Polymer (Thermo Scientific, USA) was applied and incubated for 15 minutes at room temperature then washed four times in buffer (Biogear). One drop (40µL) DAB Plus Chromogen (Thermo Scientific, USA) was added to 2 mL of DAB Plus Substrate (Thermo Scientific, USA) and mixed by swirling and applied to tissue. The substance was incubated for 5 minutes then washed 4 times in Aquabidest (PT Ikaphamindo Putramas, Jakarta, Indonesia). Counterstaining and coverslip placement was done using a permanent mounting media.

### Variables and data collection

**Figure 2:**
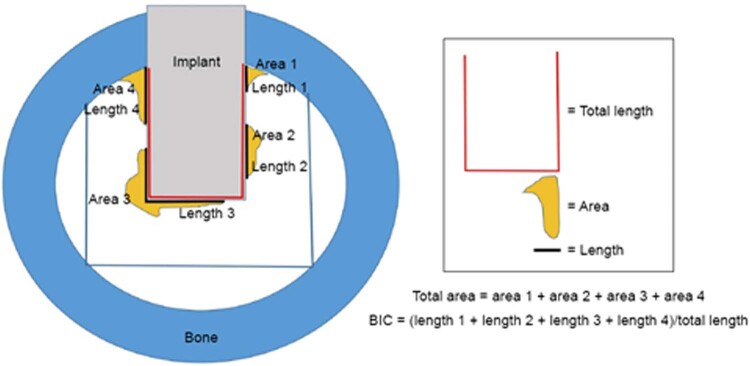
The location around outer implant used to measure bone-to-implant contact (BIC) value.47

Immunohistochemistry examination was performed for Runx2 and Osx, while histological examination was performed for osteoblast and calculated microscopically. The images were taken using a Nikon Eclipse Ci-E compound microscope equipped with a DS-Fi3 digital camera with an image resolution of 2880 × 2048 pixels. This microscope has a Tube F.O.V (field of View) 22 mm. Images were obtained using a 10× eyepiece and a 40×objective lens (400×magnification). The immunolabeled osteoblast cells were counted manually under 400× magnification. The observation area was five random fields of view under 1000 µm radius around the implant. BIC value was obtained by dividing the total length of the implant area (µm) by the length of the ossification area with a light microscope and a calibrated micrometre using 40× magnification. BIC value was obtained from the outer area of implant ( Figure 2 ).

### Statistical analysis

Normality of data was performed with the Shapiro Wilk test. Data was analysed with one-way ANOVA and continued with the Multiple Comparison LSD test at 0.05 significance level.

## Results

All animals induced with STZ intraperitoneally for five consecutive days presented hyperglycemia (> 300 mg/dL) after the fifth day (Table 10. About 10% of the rats treated with STZ (4/32) died after induction. Furthermore, randomization was carried out before implant placement and hUCMSCs injection ( Table 1 ). Blood Glucose Level was monitored on each animal before implant placement and every week until termination day. The results showed that the target hyperglycemia for diabetic animal model was maintained > 300 mg/dL ( Table 2 ).

### Immunohistochemistry examination

#### Runt-Related Transcription Factor 2 (RUNX2)

**Figure 3:**
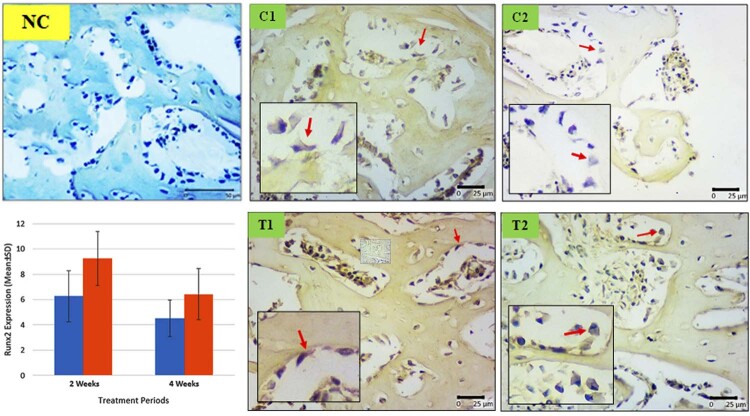
Differences of Runx2 expression in osteoblast cells between groups. Immunoreactive of surface osteoblast cells colored dark brown chromogen (arrow) (C1: implant 2 weeks, C2 : implant 4 weeks, T1 : implant + hUCMSCs 2 weeks, T2 : implant + hUCMSCs 4 weeks, NC : negative control without antibody, G : Graphics of Runx2 between groups)

Figure 3 shows the comparison of Runx2 expression among groups. The lowest Runx2 expressions were found in the C2 group (4.51 ± 1.4), while the most abundant expressions were on T1 group (9.26 ± 2.13). C1 and T2 group was having similar number of expressions, 6.26 ± 2.02 and 6.43 ± 2.01, respectively. Significant difference was found between C1 and T1 group (p < 0.01), C2 and T1 group (p < 0.001), T1 and T2 group (p < 0.05).

## Osterix

**Figure 4:**
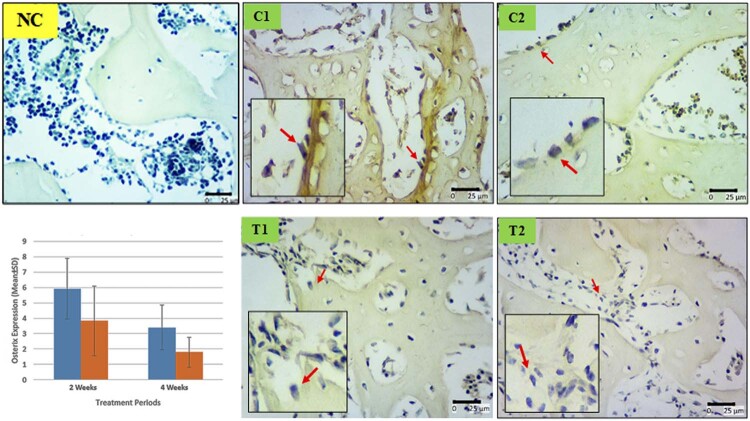
Differences of Osx expression in osteoblast cells between groups. Immunoreactive of surface osteoblast cells colored dark brown chromogen (arrow) (C1 : implant 2 weeks, C2 : implant 4 weeks, T1 : implant + hUCMSCs 2 weeks, T2 : implant + hUCMSCs 4 weeks, NC : negative control without antibody, G : Graphics of Osx between groups)

Figure 4 shows immunohistochemistry staining of Osx among groups. The highest amount of Osx expressions was found in C1 group (5.91 ± 1.97), while the lowest amount was found in T2 group (1.8 ± 0.97). T2 group differed significantly from T1 groups (3.8 ± 2.26) with p-value < 0.05. C1 group differed significantly from all other groups (p < 0.05). For bone remodelling, generally the highest expression of Osx was found in two weeks. The injection of hUCMSCs decreased the Osx expression afterwards.

## Osteoblast

**Figure 5:**
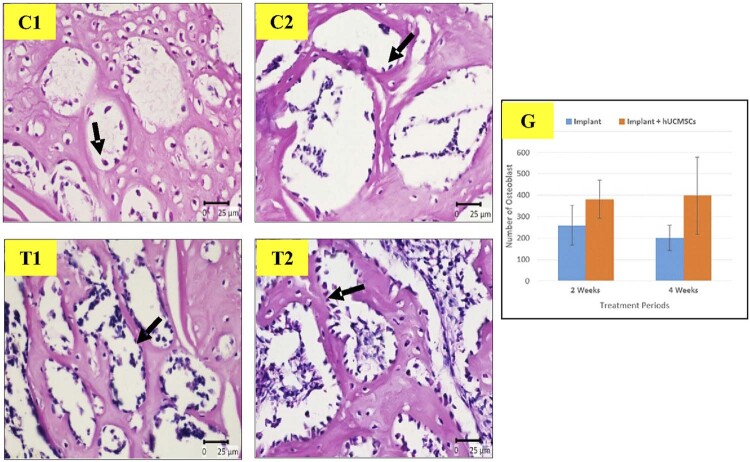
The microscopic appearance of osteoblasts between groups (C1 : implant 2 weeks, C2 : implant 4 weeks, T1 : implant + hUCMSCs 2 weeks, T2 : implant + hUCMSCs 4 weeks, G : Graphics of osteoblasts between groups)

Figure 5 shows the histological examinations. Treatment group leads the highest number of expressions with T1 group represented 381.57 ± 89.24 and T2 group represented 397.86 ± 181.8, meanwhile the control group remains low (C1 group: 259.29 ± 92.47, C2 group: 201.29 ± 59.13). The number of osteoblasts between the control and treatment groups were significantly different (C1 and T2, p < 0.05; C2 and T1, p < 0.01), C2 and T2, p < 0.01), but was found no significant difference between the control groups (C1 and C2) and between the treatment groups (T1 and T2). The lowest number of osteoblasts was found in C2, while the highest was in T2.

## Bone implant contact (BIC)

**Figure 6:**
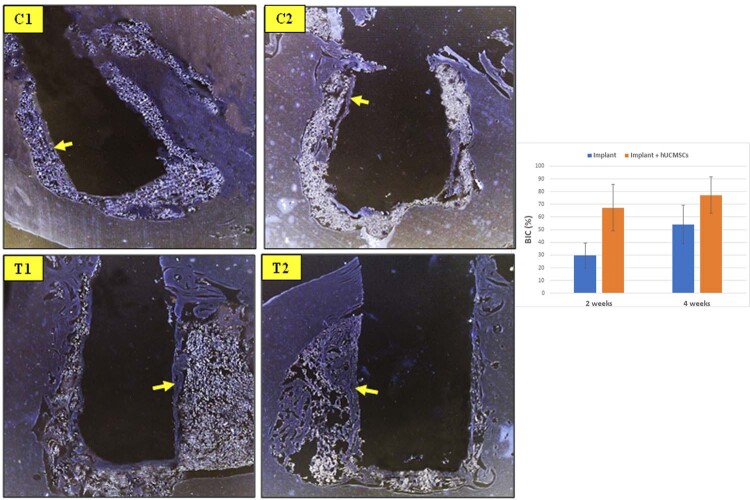
The microscopic appearance of Bone Implant Contact between groups (C1 : implant 2 weeks, C2 : implant 4 weeks, T1 : implant + hUCMSCs 2 weeks, T2 : implant + hUCMSCs 4 weeks, G : Graphics of BIC between groups)

Figure 6 shows (BIC) among groups. The highest BIC was found in T2 group (77.29% ± 14.29%), and the lowest in C1 group (29.71% ± 9.99%). The C2 and T1 group mean value of BIC length was 54% ± 15.36% and 67.29% ± 18.35%, respectively. Significant difference was found between C1 and C2 group (p < 0.01), C1 and T1 (p < 0.001), C1 and T2 (p < 0.000), and C2 and T2 (p < 0.01). No significant difference was found between treatment groups.

## Discussion

The long-term success of implants is influenced by osseointegration, which indicates the interaction between bone tissue and implant body.^8 , 21 , 22^ In uncontrolled diabetic patients, the osseointegration process of implants can be disrupted, because in a hyperglycaemic state pro-inflammatory mediators and RANKL/OPG ratio increase, while the formation and function of osteoblasts, and activity of endogenous Mesenchymal Stem Cells (MSCs) decrease.^8 , 23 - 25^

Diabetic conditions also affect the amount and function of the endogenous MSCs, caused by the accumulation of Advanced Glycation End products (AGEs).^26^ When endogenous MSCs are insufficient due to a systemic disease, administration of exogenous MSCs can be an alternative treatment. *In vitro* studies have shown that exogenous MSCs can reach wound areas that require healing, promote tissue regeneration, and improve the microenvironment around the wound. MSCs originating from the umbilical cord should be one of the therapies to improve and accelerate the osseointegration process of dental implants in diabetics because they can proliferate, differentiate, and have immunomodulatory properties.^27^ In the previous study, the induction of hUCMSCs increased osteoblastic activity, decreased osteoclastic activity, and promoted osteogenic differentiation and bone formation.^13^

In this study, hUCMSCs remained viable during the procedure. This result was also similar to previous study by Hendrijantini, et al.^45^ (2021), which showed that exogeneous hUCMSCs were detected strongly at four weeks, and even after 8 weeks. Moreover, there was some other possibilities towards hUCMSCs fate as therapeutic agent: (1) It may have been proliferated and differentiated into other cells, considering the time frame (more than two weeks);^28^ (2) MSCs provide a therapeutic effect *in vivo* via paracrine action, in particular the shedding of extracellular vesicles including exosomes and microvesicles, which secretes a variety of soluble factors to exert immunomodulatory, angiogenic, antiapoptotic, and antioxidative effects;^29^ (3) Replacement of the damaged tissue by differentiating into various cell lineages and regulation of immune responses by immunomodulatory function, (4) Cell–cell contact enables MSCs to modulate their immunosuppressive effects and promote cell viability,^30^and (5) MSCs can also increase PGE2 secretion that drives resident macrophages with an M1 proinflammatory phenotype toward an M2 anti-inflammatory phenotype, which produce higher amounts of the anti- inflammatory cytokine IL-10 and contribute to inflammation resolution.^31 , 32^

To determine the success of the dental implant osseointegration process, several markers that play an important role can be used, such as Runx2, Osx, osteoblasts, and BIC.^22 , 33^ Runx2 is a transcription factor that plays a role in skeletal growth, osteoblast differentiation, and osteoblastic lineage. The amount of Runx2 increase in immature pre-osteoblasts and osteoblasts and decrease when osteoblast maturation occurs.^34 , 35^In MSCs, Runx2 expression is weak in undifferentiated MSCs, but increases in pre-osteoblasts and reaches maximum amount in immature osteoblasts, eventually decreasing as osteoblasts mature. Runx2 also has an important role in increasing the amount of MSCs.^33^

The role of Runx2 in remodelling can be directly or via Runx2-related signalling pathways, such as Osterix.^36^The direct pathway has an involvement of Bone Morphogenetic Protein (BMP) that plays a role in controlling the differentiation of MSCs from progenitors to Runx2.^37^ Patients with type 2 diabetes mellitus showed a decrease in the amount of Runx2 expression and osteocalcin by 40%. Thus, this condition significantly decreases the bone volume, density, and trabecular bone volume.^38^

The results showed that there was no significant difference between groups C1, T1 and T2; however, in group C2 the number of Runx2 expressions was minimal. The absence of differences in Runx2 expression in groups T1 and T2 may indicate that the maximum number of Runx2 may have been reached before the end of two weeks. Thus, in groups T1 and T2 the amount of Runx2 expressions did not differ statistically. This is also supported by the BIC data that shows high contact between implants and bone in the treatment group. BIC percentage from the treatment group has fulfilled the minimum BIC required for implant success, which ranges from 50% to 80%.^39^ However, the amount of Runx2 was still high in group C1 and decreased significantly in group C2, indicating that the maturation process is likely to occur between two and four weeks. Therefore, the injection of hUCMSCs accelerates the osseointegration process of the implant. Enough Runx2 is still required to regulate osteocalcin expression and inhibit MSCs differentiation into adipogenic pathways as a result of hyperglycaemic conditions.^37^

Osterix is a transcription factor expressed on osteoblasts and required for osteoblast differentiation and maturation.^36^ In animal studies, Osx deficiency results in the absence of osteoblasts and bone formation. Osx transcriptional regulation is regulated by Runx2, and together with Nuclear Factor of Activated T cells (NFAT) activate bone formation via activation of Collagen type 1 Alpha 1 (COL1A1).^38^ In MSCs osteoblastogenesis, Osx has an important role together with Bone Morphogenic Protein (BMP) signalling, which initiates osteoblast maturation. However, overexpression of Osx can also trigger the differentiation and activation of cytokines such as IL-8 and Parathyroid Hormone-related Protein (PTHrP), which activate the osteoclastogenesis pathway.^36^ Overexpression and knockdown of Osx decrease MSCs proliferation, thus Osx plays an important role in the MSCs’ proliferation process at different stages of differentiation. The stem cells source also influences the effects of Osx. In MSCSs derived from rats, Osx increase the proliferation of bone marrow stromal cells. On the other hand, if the MSCs are from humans, Osx inhibits cell growth and cause excessive mineralization in experimental animals.^40^

In this study, the maturation process of the treatment group with hUCMSCs was faster than that of the control group, since the amount of Osx in the C2 group was the same as in the T1 group, followed by a decrease in the Osx in the T2 group. This is also supported by the results of the higher BIC in the treatment group than in the control group. Moreover, as the source of MSCs is human, higher amounts of Osx result in a negative role in the cell proliferation process and trigger excessive mineralization process.

The bone remodelling cycle runs well if there is a balance between the process of bone formation by osteoblasts and the resorption by osteoclasts.^40^ Osteoblasts are bone-forming cells derived from MSCs after passing by several transcription factors such as BMP and Wnt pathways *.* An increase in the number of osteoblasts also increases the amount of Osterix, and decreases the amount of RANKL, the ratio of RANKL/OPG, and the expression of cathepsin K. Furthermore, high amount of osteoblasts suppresses the production of TNF, which acts in the process of resorption and osteoclastogenesis.^40^ In patients with type 2 diabetes mellitus, the volume and thickness of the osteoid, the amount of osteoblasts, and Alkaline Phosphatase (ALP) decrease. Osteoblasts in high glucose concentrations cause less pro-osteogenic markers such as Runx2 and Osx.^41^ Moreover, apoptosis of osteoblasts and their precursor cells increased.^39^ Type 2 diabetes mellitus also increases the negative effect on osteoblasts by directing the differentiation of MSCs into adipose, leading to low osteoblast function, formation, and bone mass.^42^

This study showed the highest number of osteoblasts in T2 and the lowest in C2. The number of osteoblasts decreased in the control groups C1 and C2, but we found no significant difference between the treatment groups T1 and T2. hUCMSCs can increase the number of osteoblasts and the expression of the pro-osteogenic marker Runx2. hUCMSCs also inhibited the process of osteoblast apoptosis and increased osteoblast activity, since BIC was higher in the treatment group than in the control group. A constant number of osteoblasts is essential in long-term implant osseointegration by internal and external bone remodelling.^22^

Bone density also has an important role in achieving the minimum required BIC. Implants placement with the same osteotomy and prosthetic procedures showed different success rates due to different types of bone density.^43^ The prognosis for implant success are higher in the anterior mandible, which has the highest density compared to the posterior mandible, while failure is mostly found in the posterior maxillary placement.^44^ In an osteoporosis model study, MSC successfully increased mandibular bone density.^45^

In dental implant osseointegration, secondary implant stability is also an important factor for long-term implant success. BIC plays an important role in the establishment of secondary implant stability. BIC is a histomorphometry examination that was developed and is a commonly used method for evaluating osseointegration. After the minimum BIC is achieved, measurement of the bone implant volume (BIV), or the area of ossification around the implant, can be performed, Thus, long-term osseointegration was successful by calculating the thickness of the new bone formed. BIC and BIV have a close correlation for the evaluation of implant osseointegration.^21^ According to Wolff’s law, when an implant is placed in the jawbone, its microenvironment changes and internal structure in response to implant placement and loading. Clinically, the minimum BIC required for implant success is from 50% to 80%.^39^

The previous study in diabetic patients reports a delay in the process of bone formation and remodelling. Studies using experimental diabetic rats terminated 2 weeks after implantation, showing an average BIC of 28.82% and 56.55% for non-diabetic rats. After 6 weeks, the studies showed an average BIC of 51% for diabetic rats and 66.4% for non-diabetic rats. In this study, local infiltration with insulin was given to the implant site with an average BIC for 2 weeks of 50.73% and 58.3% for 6 weeks, both significantly lower when compared to the non-diabetic group.^42^

The use of Nerve Growth Factor to increase BIC in diabetic experimental animals showed significant results with termination times of two, four, and eight weeks. In the two week data analysis, the mean BIC was 36.97% for the non-diabetic group, 22.11% for the diabetic group, and 36.97% for the NGF group. For the four week group, BIC increased 55.46% for the non-diabetic group, 42.61% for the diabetes group, and 54.34% for the group with NGF administration. Meanwhile, for the six week group the average BIC was 65.44% for the non-diabetic group, 55.75% for the diabetes group, and 67.99% for the group receiving NGF.^46^

This study observation lasted up to 4 weeks. For future studies, we suggest adding more time interval, thus the peak level of each expression can be known and the exact mechanism can be revealed. In this study, we also did not compare the use of insulin as a control positive group. Many factors were unknown and need to be further explored. Hopefully, this study can be a start for future development of hUCMSCs as a treatment for dental implant osseointegration under diabetes mellitus circumstances.

In this study, the highest BIC was in the T2 group and the lowest was in the C1 group. The mean BIC data for the C1 and C2 (30% and 54 %) are almost similar with the data from previous studies on implants in diabetic experimental animals.^42^ For stem cells treatment groups, hUCMSCs increased the mean BIC to 67% and 77%. Compared to other studies such as local insulin injection and NGF administration, hUCMSCs could potentially accelerate and increase BIC. Moreover, hUCMSCs increased the two weeks and four weeks BIC in diabetic animal higher than in non-diabetic group.

## Conclusion

hUCMSCs successfully accelerated and increased dental implant osseointegration in diabetic condition regarding Runx2, Osterix, osteoblasts, and BIC at two and four weeks examination.

## References

[1] Ikimi NU, Sorunke ME, Onigbinde OO, Adetoye JO, Amrore I, O Jacob O (2017). A study of the relationship between diabetes mellitus and tooth loss among diabetic patents in Garki General Hospital Garki Abuja, Fct Nigeria. Dentistry.

[2] Greenblatt AP, Salazar CR, Northridge ME, Kaplan RC, Taylor GW, Finlayson TL (2016). Association of diabetes with tooth loss in Hispanic/Latino adults: findings from the Hispanic Community Health Study/Study of Latinos. BMJ Open Diabetes Res Care.

[3] Brennan DS, Spencer AJ, Roberts-Thomson KF (2008). Tooth loss, chewing ability and quality of life. Qual Life Res.

[4] Jivraj S, Chee W (2006). Rationale for dental implants. Br Dent J.

[5] Hasegawa H, Ozawa S, Hashimoto K, Takeichi T, Ogawa T (2008). Type 2 diabetes impairs implant osseointegration capacity in rats. Int J Oral Maxillofac Implants.

[6] Alghamdi HS (2018). Methods to improve osseointegration of dental implants in low quality (type-iv) bone: an overview. J Funct Biomater.

[7] Javed F, Ahmed HB, Crespi R, Romanos GE (2013). Role of primary stability for successful osseointegration of dental implants: factors of influence and evaluation. Interv Med Appl Sci.

[8] Naujokat H, Kunzendorf B, Wiltfang J (2016). Dental implants and diabetes mellitus-a systematic review. Int J Implant Dent.

[9] Zarrabi M, Mousavi SH, Abroun S, Sadeghi B (2014). Potential uses for cord blood mesenchymal stem cells. Cell J.

[10] Qin HL, Zhu XH, Zhang B, Zhou L, Wang WY (2016). Clinical evaluation of human umbilical cord mesenchymal stem cell transplantation after angioplasty for diabetic foot. Exp Clin Endocrinol Diabetes.

[11] Hansmann G, Chouvarine P, Diekmann F, Giera M, Ralser M, Mülleder M (2022). Human umbilical cord mesenchymal stem cell-derived treatment of severe pulmonary arterial hypertension. Nat Cardiovasc Res.

[12] Zhang Q, Huang K, Lv J, Fang X, He J, Lv A (2021). Case report: human umbilical cord mesenchymal stem cells as a therapeutic intervention for a critically ill COVID-19 patient. Front Med (Lausanne).

[13] Hendrijantini N, Kusumaningsih T, Rostiny R, Mulawardhana P, Danudiningrat CP, Rantam FA (2018). A potential therapy of human umbilical cord mesenchymal stem cells for bone regeneration on osteoporotic mandibular bone. Eur J Dent.

[14] Kuntjoro M, Prasetyo EP, Cahyani F, Kamadjaja MJK, Hendrijantini N, Laksono H (2020). Lipopolysaccharide’s cytotoxicity on human umbilical cord mesenchymal stem cells. Pesqui Bras Odontopediatria Clin Integr.

[15] Prasetyo EP, Kuntjoro M, Goenharto S, Juniarti DE, Cahyani F, Hendrijantini N (2021). Calcium hydroxide increases human umbilical cord mesenchymal stem cells expressions of apoptotic protease-activating factor-1, caspase-3 and caspase-9. Clin Cosmet Investig Dent.

[16] Fang S, Liu Z, Wu S, Chen X, You M, Li Y (2022). Pro-angiognetic and pro-osteogenic effects of human umbilical cord mesenchymal stem cell-derived exosomal miR-21-5p in osteonecrosis of the femoral head. Cell Death Discov.

[17] Prasetyo EP, Widjiastuti I, Cahyani F, Kuntjoro M, Hendrijantini N, Hariyani N (2020). Cytotoxicity of calcium hydroxide on human umbilical cord mesenchymal stem cells. Pesqui Bras Odontopediatria Clin Integr.

[18] Hendrijantini N, Kresnoadi U, Salim S, Agustono B, Retnowati E, Syahrial I (2015). Study Biocompatibility and osteogenic differentiation potential of Human Umbilical Cord Mesenchymal Stem Cells (hUCMSCs) with gelatin solvent. J Biomed Sci Eng.

[19] Siniscalco D, Trotta MC, Brigida AL, Maisto R, Luongo M, Ferraraccio F (2018). Intraperitoneal administration of oxygen/ozone to rats reduces the pancreatic damage induced by streptozotocin. Biology (Basel).

[20] Han JM, Hong G, Lin H, Shimizu Y, Wu Y, Zheng G (2016). Biomechanical and histological evaluation of the osseointegration capacity of two types of zirconia implant. Int J Nanomedicine.

[21] Bernhardt R, Kuhlisch E, Schulz MC, Eckelt U, Stadlinger B (2012). Comparison of bone-implant contact and bone-implant volume between 2D-histological sections and 3D-SRµCT slices. Eur Cell Mater.

[22] Mello AS, Santos PL, Marquesi A, Queiroz TP, Margonar R, Faloni AP (2018). Some aspects of bone remodeling around dental implants. Rev Clin Periodoncia Implantol Rehabil Oral.

[23] Catalfamo DL, Britten TM, Storch DI, Calderon NL, Sorenson HL, Wallet SM (2013). Hyperglycemia induced and intrinsic alterations in type 2 diabetes-derived osteoclast function. Oral Dis.

[24] Jiao H, Xiao E, Graves DT (2015). Diabetes and its effect on bone and fracture healing. Curr Osteoporos Rep.

[25] Okonkwo UA, DiPietro LA (2017). Diabetes and wound angiogenesis. Int J Mol Sci.

[26] Kuntjoro M, Agustono B, Prasetyo EP, Salim S, Rantam FA, Hendrijantini N (2021). The effect of Advanced Glycation End products (AGEs) on human Umbilical Cord Mesenchymal Stem Cells (hUCMSCS) with regard to osteogenesis and calcification. Res J Pharm Technol.

[27] Ding DC, Chang YH, Shyu WC, Lin SZ (2015). Human umbilical cord mesenchymal stem cells: a new era for stem cell therapy. Cell Transplant.

[28] Hu Y, Zhang Y, Ni CY, Chen CY, Rao SS, Yin H (2020). Human umbilical cord mesenchymal stromal cells-derived extracellular vesicles exert potent bone protective effects by CLEC11A-mediated regulation of bone metabolism. Theranostics.

[29] Fan XL, Zhang Y, Li X, Fu QL (2020). Mechanisms underlying the protective effects of mesenchymal stem cell-based therapy. Cell Mol Life Sci.

[30] Fan XL, Zhang Y, Li X, Fu QL (2020). Mechanisms underlying the protective effects of mesenchymal stem cell-based therapy. Cell Mol Life Sci.

[31] Jahromi SH, Estrada C, Li Y, Cheng E, Davies JE (2018). Human Umbilical Cord Perivascular Cells and Human Bone Marrow Mesenchymal Stromal Cells Transplanted Intramuscularly Respond to a Distant Source of Inflammation. Stem Cells Dev.

[32] Braza F, Dirou S, Forest V, Sauzeau V, Hassoun D, Chesné J (2016). Mesenchymal stem cells induce suppressive macrophages through phagocytosis in a mouse model of asthma. Stem Cells.

[33] Komori T (2019). Regulation of proliferation, differentiation and functions of osteoblasts by Runx2. Int J Mol Sci.

[34] Mevel R, Draper JE, Lie-A-Ling M, Kouskoff V, Lacaud G (2019). RUNX transcription factors: orchestrators of development. Development.

[35] Chen S, Gluhak-Heinrich J, Wang YH, Wu YM, Chuang HH, Chen L (2009). Runx2, osx, and dspp in tooth development. J Dent Res.

[36] Liu Q, Li M, Wang S, Xiao Z, Xiong Y, Wang G (2020). Recent Advances of osterix transcription factor in osteoblast differentiation and bone formation. Front Cell Dev Biol.

[37] Wallner C, Schira J, Wagner JM, Schulte M, Fischer S, Hirsch T (2015). Application of VEGFA and FGF-9 enhances angiogenesis, osteogenesis and bone remodeling in type 2 diabetic long bone regeneration. PLoS One.

[38] Zhu F, Friedman MS, Luo W, Woolf P, Hankenson KD (2012). The transcription factor osterix (SP7) regulates BMP6-induced human osteoblast differentiation. J Cell Physiol.

[39] Lian Z, Guan H, Ivanovski S, Loo YC, Johnson NW, Zhang H (2010). Effect of bone to implant contact percentage on bone remodelling surrounding a dental implant. Int J Oral Maxillofac Surg.

[40] Kenkre JS, Bassett J (2018). The bone remodelling cycle. Ann Clin Biochem.

[41] Picke AK, Campbell G, Napoli N, Hofbauer LC, Rauner M (2019). Update on the impact of type 2 diabetes mellitus on bone metabolism and material properties. Endocr Connect.

[42] Wang B, Song Y, Wang F, Li D, Zhang H, Ma A (2011). Effects of local infiltration of insulin around titanium implants in diabetic rats. Br J Oral Maxillofac Surg.

[43] Mahmoud NS, Mohamed MR, Ali MAM, Aglan HA, Amr KS, Ahmed HH (2020). Osteoblast-based therapy-a new approach for bone repair in osteoporosis: pre-clinical setting. Tissue Eng Regen Med.

[44] Misch CE (2008). Contemporary implant dentistry.

[45] Hendrijantini N, Hartono CK, Daniati RP, Hong G, Sitalaksmi RM, Kuntjoro M (2021). Human umbilical cord mesenchymal stem cell-induced osterix, bone morphogenetic protein-2, and tartrate-resistant acid phosphatase expression in osteoporotic mandibular bone. Eur J Dent.

[46] Zhang J, Shirai M, Yamamoto R, Yamakoshi Y, Oida S, Ohkubo C (2016). effect of nerve growth factor on osseointegration of titanium implants in type 2 diabetic rats. Int J Oral Maxillofac Implants.

[47] Han JM, Hong G, Lin H, Shimizu Y, Wu Y, Zheng G (2016). Biomechanical and histological evaluation of the osseointegration capacity of two types of zirconia implant. Int J Nanomedicine.

